# Indents

**Published:** 1903-12-15

**Authors:** 


### INDENTS.

**☞ Brief Articles of Technical or Scientific Value will receive notice and comment. Contributions invited.**

**Adapting Logan Crowns.** Dr. J. D. Patterson showed at the meeting of the National Dental Association a very neat method of adapting Logan crowns. The root end is dressed to desired form, below the gum margin on the labial face so as to hide the joint, and a disc of platinum burnished to an exact adaptation. A selected Logan crown is then ground on the lingual aspect so as to leave a free V-shaped opening. The crown post is then forced through the platinum disc to position and the V-shaped space filled with adhesive wax. The crown is then removed from the root bringing the platinum cap with it. This is invested in suitable material, the wax removed and the crown and cap attached with porcelain. The result is a well-fitted, strong and esthetic crown.—Dr. Guilford, in *Stomatologist.*

**Neutral Soap.** M. P. Dieterich gives the following process for preparing a perfectly neutral soap:

Solution Caustic Soda (sp. gr. 1.168-1.172)............130 Gm.
Heat on a steam bath, and add gradually a melted mixture of
Lard.................................................50 Gm.
Olive Oil............................................50 Gm.
Boil for half an hour under constant stirring, and then add
Alcohol (90 percent)...................................12 Gm.
As soon as the mass has become uniform, dilute it with
Water...............................................130 Gm.
Heat again, and then add a filtered solution of
Sodium Chloride....................................37.5 Gm.
Sodium Carbonate....................................4.5 Gm.
Water...............................................120 Gm.

The heat is continued until the soap has entirely separated, when it is removed after cooling, washed with a little water, and then carefully yet forcibly pressed. Medicinal substances or perfumes may then be added, if desired, in the requisite quantities.—*Merck's Report.*

**Root Filling Material.** Gutta-Percha base plate, weight ½ oz. Saturate solution of thymol in eucalypti, measure ½ oz. Dissolve gutta-percha in chloroform, add thymol and eucalypti and mix thoroughly. Set aside to allow chloroform to evaporate.

In manipulating the above, after the tooth is dry place some of the filling in the pulp chamber and with a warm broach work it into the canals. It can then be forced to the apex with a soft piece of rubber, and the gutta-percha cone inserted into the usual way. If the canal is too small to admit a cone, a fiber of cotton may be used, or if too small for cotton, the filling can be forced into the warm canal with soft rubber, which is far better than no filling. I believe this to be much better than chloro-percha, for two reasons, viz: the presence of thymol and eucalypti, and no change in the material shape. After nearly five years' test in the mouth, I have found a distinct odor of the thymol and eucalypti.—B. L. Cochran, in *Review.*

**Obtunding a tooth in Preparation for a Crown.** Dr. J. L. Young, of Detroit, gives this method: Expose some of the dentine preferably on the occlusal surface of the tooth. This is easily and almost painlessly done with a knife-edged carborundrum disk (kept wet) in the engine. Apply dam and thoroughly insulate, so that current can not pass between neck of tooth and rubber. Chloro-percha is efficient for this, and if a clamp has to be used, it should be thoroughly coated with chloro-percha. With negative electrode in the patient's hand and positive carrying a small pellet of cotton, moistened with a saturated solution of cocain placed in contact with dentine, instruct the patient to gradually turn on the current with the other hand. This will usually take from five to ten minutes to get the controller up to one hundred points. It is then left at this point for ten or fifteen minutes, or until such time as the positive electrode can be removed from the tooth and replaced without causing pain. Then remove dam and grind the tooth to shape, which usually requires that all the enamel must be removed.

Readjust dam and coat the tooth with silver nitrate, closing up the dental tubuli, thus rendering pulp immune to thermal shock and action of acid in cement.—*Review.*

**A Business Method.** Just now the business side of dental practice is claiming quite a little attention. All who tackle the subject start with the aphorism that as a rule professional men are bad business men. True, but not this one: He is an "aside" as to location, but has just notions of what is right and wrong; that what is sauce for the goose is also good sauce for the gander. He figures his time worth two dollars an hour. If the patient is half an hour late he must pay for the half hour before the doctor deigns to begin work. It must be *paid* then and there; that's for time, not dentistry. That part settled, he goes on with the work for which the patient specially engaged him. Conversely, if by any chance the doctor is behind, say a quarter of an hour, he hands the patient half a dollar before beginning the service. He has done this from the first, and to-day his methods is so well understood that the patient belated knows just what is expected of him and just what he must do in order to be waited upon. He knows, too, that if the doctor is delayed in beginning his part, there will be a settlement for time forthwith. We haven't the honor of personal acquaintance with the gentleman, but apples to doughnuts he has a bank account and all the credit he may have occasion to use.—*Dental Office and Laboratory.*

**How to Make Gold Crowns.** Prepare tooth or root in the usual way. Take measure with wire, cut band, one-sixty-fourth of an inch longer than the measure; bevel edges, lap, apply borax solution, and sweat together in Bunsen flame; try on root, cut to fit gum margin, contour with pliers, try on again, and observe articulation; trim top of band to allow free occlusion of the teeth with band in position on root, also lateral motion of jaw. Next heat over flame a piece of impression compound, place in top of band and have patient bite, holding teeth together half a minute till hard; remove band with the compound in it, carve up for cusps, leaving articulating surface, as shown on compound; pour some plaster on a glass and place crown band with compound top into it; when hard, remove. A small taper ferrule is now driven over the impression in the plaster and fusible metal poured in; take die thus obtained, and strike up top for crown on piece of lead; fit this over the crown band with compound still in it; trim, and mark with a file edge of both top and band; now remove compound from band; clean both top and band in acid; put top on, having marks together; use twisted wire around tweezers, which holds them together, solder from inside with Bunsen flame, filling cusps at the same time; polish and cement on. Articulation is perfect. This can be made and cemented on in one hour.—Dr. O. Martin, in *Review*.

**Pyorrhea a Case.** A lady about forty, presented with a swelling on the labial gum, near the apex of a lower right lateral. Exploration revealed the fact that there was a pocket filled with pus on that surface of the tooth, which extended to the mesial surface. The labial bone had all disappeared, although the gum had not receded. A dentist sent her to me, saying he could do nothing more for her and he thought the tooth would have to be extracted. He had treated it for "pyorrhea" for about six months, but the condition gradually grew worse and the tooth became loose and separated from the central badly.

Careful exploration revealed the fact that those surfaces of the root were covered with small white nodular deposits, very hard. A cross incision was made in the gum through which the deposits were removed. The case was treated with glycerol of idoine and zinc every third day for two weeks then twice a week for a month; then once a week for another month. An antiseptic astringent mouth-wash was prescribed and daily massage of the tissues around the tooth was directed.

In three months' time recovery was complete. The tooth had moved back into normal position without other assistance, and bone formed on the labial of the tooth where it had been absorbed. At the present time, one year since treatment was completed, I do not think any one can discover which tooth was affected. The only unusual thing about this case was the rapid formation of bone over the tooth root. It had been absorbed and discharging pus for over three years, and is a vital tooth. Try this line of treatment for your next case of pyorrhea, and don't say it can't be cured.—E. MaWhinney, in *North-Western Journal.*

**Removing Impacted Third Molar.**

In removing these teeth I usually incise the soft tissue which covers the tooth or in any way tends to retard its removal. To do this I prefer to use a pair of sharp scissors which crushes the vessels together and causes less bleeding than the knife. The use of the scissors lessens the danger of sepsis, by preventing the pus or clots being carried into the vessels. I next remove with suitable burs and dental engine the bone which holds down the tooth. Here the greatest care must be observed lest the soft tissues on the lingual surface be disturbed, as paralysis of the tongue is liable to follow such a wound. I aim to keep within the periosteum, except that portion which is removed with the bone lying over the tooth. When this is accomplished removing the tooth is the next step and is by no means the easiest part of this perplexing operation. The Physick forceps and the elevator are generally employed. In most cases I find the elevator better adapted. When the Physick forceps are used the second molar must form a fulcrum upon which to pry the tooth. When the elevator is used a depression is drilled into the bone under the front portion of the tooth at a proper angle so that when it is forced under the tooth it can better be raised from its bed. When the bone about the tooth is necrosed or carious it should be burred or curetted away, and when the wound is cleansed of the debris it should be tightly packed with antiseptic gauze, not alone for the purpose of preventing sepsis but for checking hemorrhage. This packing may be removed on the following day. Thereafter the wound should be lightly packed so as to preserve cleanliness and facilitate granulation, until the cavity in the jaw is filled and the wound healed. It must be borne in mind that when a wound is tightly packed granulations will no more form than will grass grow under the pressure of a board.—Dr. L. Curtis, in *Review.*

**Fusion, Shrinkage and Strength of Porcelain.**

Dr. Le Cron, in the November *Summary,* contributes a valuable article to porcelain manipulation, in which he says "It is of the utmost value to know the true fusion or baking point. If underbaked the porcelain will be brittle and flake and crush under abnormal pressure. If overbaked it becomes porous and weak. The three methods of watching for a certain heat, testing with pure gold, or timing the furnace, are all more or less unreliable; because of the blinding light, the only approximate melting point of gold, and the unsteady power of electric currents. One can use pure gold and get approximate fusion and by timing the current and observing the light, get fairly good results, but not always accurate ones.

To ascertain the true and comparative fusing points of different porcelains, I made tests by use of the Seger Cones and a thermo-electric pyrometer, and testing the results at varying fusing points, I think I have established the temperature at which each body fuses to produce thestrongest and best results, as shown in the following table:

| | Fahrenheit. | | Fahrenheit |
|---|---|---|---|
| Allen's | 2340° | Consolidated Inlay | 2138° |
| Close's | 2288° | Whitely | 2138° |
| White's Inlay | 2254° | Brewster Enamel | 2084° |
| Brewster's Foundation | 2218° | Ash High-Fusing | 2012° |
| Consolidated Con. Gum | 2200° | Jenkins | 1580° |

I am satisfied they are absolutely correct, for the most minute attention was given to every detail.

Experiments will show that correct fusing of porcelain, to reach the highest point of strength, is absolutely necessary; and I may add that much of success in this line of work depends on a knowledge of the same.

The crushing strength will be shown in the following table:

| Name of Porcelain. | Break. Load. pounds. | Crush. St'ngth Lbs. per sq. in |
|---|---|---|
| Allen Body | 3450 | 26950 |
| Close Body | 5650 | 45640 |
| White's Inlay Body | 3800 | 32205 |
| Brewster's Foundation | 2540 | 20320 |
| Consolidated Continuous Gum | 3860 | 30390 |
| Whitely's Body | 2110 | 16000 |
| Brewster's Enamel | 2690 | 22990 |
| Ash's High-Fusing | 2760 | 22810 |
| Jenkins' Body | 3330 | 28305 |
| Consolidated Inlay | 2100 | 15080 |

The shrinkage will be shown in the following table:

| Names of Porcelain Bodies. | Per Ct. Shrink. |
|---|---|
| Consolidated Continuous Gum Body | $21\frac{1}{2}$ |
| Close's Body | $21\frac{3}{4}$ |
| Allen's Body | $22\frac{1}{2}$ |
| White's Inlay Body | $23\frac{1}{2}$ |
| Brewster's Foundation Body | $23\frac{3}{4}$ |
| Consolidated Inlay Body | 31 |
| Whitely's Body | 31 |
| Brewster's Enamel Body | 33 |
| Ash's High Fusing Body | $34\frac{3}{4}$ |
| Jenkins' Body | $38\frac{1}{4}$ |

Clinical experience and these tests have led me to prefer Close's body for continuous gum and bridge-work, and White's body for general work, because of their greater strength, density, color, outlines and low percent of shrinkage. All these qualities are of the greatest value in porcelain work, where exactness is so necessary to success.

